# B-cell peripheral tolerance: think dynamics beyond affinity

**DOI:** 10.3389/fimmu.2026.1799465

**Published:** 2026-05-01

**Authors:** Patrick Henong Yuan, Helen Huang, Haripriya Vaidehi Narayanan, Alexander Hoffmann

**Affiliations:** 1 Institute for Quantitative and Computational Biosciences, University of California, Los Angeles, Los Angeles, CA, United States; 2 Biomathematics Graduate Program, University of California, Los Angeles, Los Angeles, CA, United States; 3 Bioinformatics Interdepartmental Program, University of California, Los Angeles, Los Angeles, CA, United States; 4 Department of Microbiology, Immunology, and Molecular Genetics, University of California, Los Angeles, Los Angeles, CA, United States

**Keywords:** Age-associated B cells, AICD, anergic T cells, antigen capture, antigen receptor, autoimmune pathologies, B1-8hi splenic B cells, B1-8lo

## Abstract

Maintaining B-cell tolerance – the elimination of self-reactive B-cells – is literally a life-long challenge because the B-cell receptor mutates to generate millions of new variants during each immune response in the germinal center (GC) of lymph nodes. B-cell tolerance therefore rests on local mechanisms within GCs, i.e., in the periphery, whereas classical T-cell tolerance mechanisms operate in the thymus (central tolerance) when T-cell diversity is generated during immune development. While T-cell tolerance has been shown to be mediated by kinetic proofreading mechanisms that are capable of distinguishing small affinity differences, we argue here that B-cell tolerance mechanisms in the GC operate at different timescales covering intra- and, inter-cellular signaling in the light zone, and iterative light-dark-zone cycling, where the temporal relationship between BCR and CD40 signaling are key to determining whether a B-cell is positively or negatively selected. This review summarizes how recent research points to a new conceptual paradigm for B-cell tolerance that must involve the analysis of intracellular and intercellular network dynamics. We show how multi-scale dynamical systems modeling will be key to elucidate B-cell selection mechanisms and enable a predictive understanding of how autoimmune pathologies arise.

## 1 Introduction

The maintenance of B-cell peripheral tolerance is uniquely challenging. Unlike T cells, which possess a fixed antigen receptor following thymic selection, the B-cell repertoire is not fixed. After activation in the germinal center (GC), B cells undergo somatic hypermutation (SHM) in their B-cell receptor (BCR) to explore the affinity space, and can generate *de novo* polyreactivity—defined as the ability to bind to multiple, structurally unrelated antigens, which can be both self and foreign, with low to moderate affinity—and self-reactivity—defined as the ability to bind to the body’s own antigen—even when the naïve starting clone was not overtly autoreactive (1, 2). Peripheral tolerance in B cells is therefore an ongoing control problem continuously challenged by the very mechanism that enables protective humoral immunity. A classic conceptual solution is the two-signal hypothesis: BCR engagement is not itself the stimulatory signal, but rather a licensing step that, through antigen uptake, processing and display, allows for subsequent cognate T-cell engagement, which provides the survival and proliferation cues (3, 4). Because T follicular helper (Tfh) cells preferentially interact with B cells that present more antigen-derived peptides, higher BCR affinity results in greater access to Tfh growth signals and larger clonal expansion, while those that fail to obtain sufficient help die by neglect (4–7).

This model accounts for peripheral tolerance through positive selection only. Autoreactive B cells are not actively removed, but simply fail to obtain the stimulatory second signal required for proliferative expansion. In other words, B-cell peripheral tolerance becomes an extension of central T-cell tolerance: the fidelity of B-cell peripheral tolerance depends on how strictly thymic selection constrains T-cell specificity during development. However, two well-established features of T cell biology complicate this picture. First, T-cell central tolerance is leaky: as many as 25-40% of self-reactive T cells escape deletion in the thymus and persist in the mature repertoire (8–11). As a result, about 4% of mature non-regulatory T cells are self-reactive, a magnitude comparable to the 1-10% that are reactive to alloantigens, which are non-self-antigens from another individual of the same species such as transplanted tissues, and the 5-20% reactive to superantigens, which are microbial proteins that activate large fractions of T cells by bypassing conventional peptide specificity (12–15). Both have been long recognized as biologically consequential classes of T-cell reactivity. Additionally, T cells are intrinsically cross-reactive, which is defined as the ability to recognize and bind with high affinity to more than one antigen due to shared molecular features. Theoretical and experimental estimates indicate that a single T-cell receptor (TCR) must be capable of recognizing approximately 10^6^ distinct peptides on average in order to provide comprehensive coverage against pathogens (16, 17). This combination of leakiness and cross-reactivity may be precisely what enables immunity against tumors, where the immunological target space overlaps with the self, but it renders the absence of T-cell help and death by neglect an unreliable safeguard for B-cell peripheral tolerance. Hence, the question remains: if potentially supportive T cells exist, and if help can be promiscuously triggered, how does B-cell peripheral tolerance hold across decades of immune response development?

To resolve this paradox, we must look beyond static affinity thresholds and consider the spatiotemporal dynamics of the GC reaction. The removal of self-reactive B cells is an emergent property of a multi-scale process that integrates the kinetics of antigen capture, the stochastic search for Tfh cells, and the processing of these signals within the B-cell intracellular molecular network (18, 19). Critically, the same inputs—BCR engagement, Tfh signaling, and cytokine exposure—can yield distinct outcomes depending on the molecular state of the B cell at the time of signal receipt and on the temporal relationships among these inputs (20–22). Small differences in fate decisions at the single-cell level can then be amplified into divergent clonal outcomes through repeated cycles of selection and proliferation. Hence, a robust model of B-cell peripheral tolerance requires bridging the gap between the intracellular signaling networks that determine individual B-cell fate, the intercellular interaction dynamics that unfold within the spatially structured GC environment, and the emergent clonal outcomes of the GC reaction.

Understanding this multi-scale control of B-cell peripheral tolerance is clinically imperative because its failure is the basis of diverse pathologies, most notably systemic autoimmunity. In conditions such as Systemic Lupus Erythematosus (SLE), the breakdown of these dynamic checkpoints allows B cells to act as potent antigen-presenting cells that activate low-affinity or anergic T cells, seeding a pathologic positive feedback loop that perpetuates disease (23). Furthermore, this dynamic control is compromised in aging, where inflammaging creates a noisy signaling environment that may lower activation thresholds and weaken the insulation of a specific Tfh-B-cell interaction, increasing the risk that self-reactive B-cells proliferate and differentiate into plasma cells, and thereby fueling late-onset autoimmunity (24, 25). While regulatory T-cells play key roles in dampening the emergence such auto-immune activity, in this minireview we focus on how multi-scale dynamics of B-cells with antigen-presenting cells and Tfh cells within the GC control the elimination of self-reactive B-cells.

## 2 Intracellular models of B-cell fate decisions: the temporal integration of BCR and CD40

B-cell tolerance requires the selective elimination of self-reactive B cells, which means that such B-cells must make a decision to undergo apoptotic cell death. There is now abundant *in vivo* evidence that both antigen-dependent BCR and Tfh-derived CD40 receptor signaling control the generation of an antibody response. CD40 signaling is unambiguously pro-survival and pro-proliferative, and disrupting CD40-CD40L signaling cripples the GC response (26, 27). However, the role of BCR signaling, besides its licensing ability, appears to be more complicated. On one hand, Chen et al. found that BCR signaling prolongs B-cell survival in the GC and primes B cells to receive the secondary Tfh signal. On the other hand, GC B cells dampen BCR signal transduction through elevated phosphatase activity (28), and multiple mouse models indicate that exaggerating BCR signaling by removing its negative regulators can be detrimental rather than beneficial to B-cell survival. Conditional loss of the inhibitory phosphatase SHP-1 enhances proximal BCR signaling, yet reduces the early GC response and increases GC B-cell apoptosis (29). Likewise, deletion of the kinase inhibitor Csk in GC B cells augments net BCR signaling strength, yet imposes a competitive disadvantage due to increased apoptotic stress compared to wild-type controls (30). In a comparative model for BCR affinity-dependent competition, higher-affinity B1-8^hi^ splenic B cells undergo both increased proliferation but also increased apoptosis relative to their lower-affinity B1-8^lo^ counterparts after immunization, underscoring again the paradoxical role of strong BCR engagement (31).

**Figure 1:**
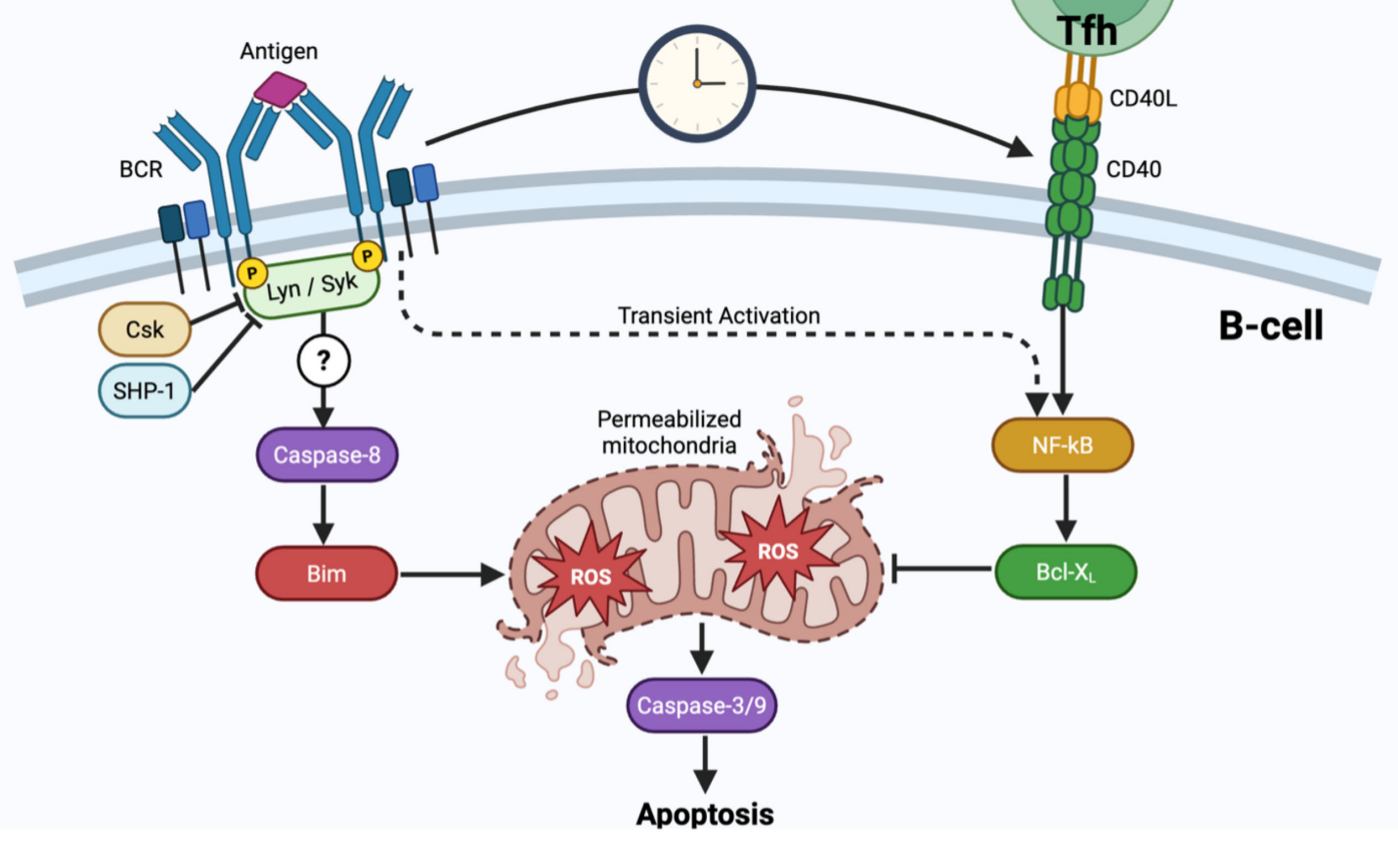
Intracellular kinetic competition between pro-death and pro-survival pathways. (Left) BCR engagement initiates a pro-death signaling cascade regulated proximally by Csk and SHP-1. This signal drives Caspase-8 activation and Bim translocation, resulting in sustained mitochondrial ROS production and membrane permeabilization. (Right) Tfh interaction (CD40-CD40L) provides a robust rescue signal by inducing sustained NFκB activation. (Center) The cell fate decision is determined at the mitochondria, where CD40-induced Bcl-xL must accumulate sufficiently to neutralize the pro-apoptotic effects of Bim and ROS. Note that BCR stimulation alone induces only transient NFκB activation, which is insufficient to counteract the time-dependent accumulation of mitochondrial death signals.

In parallel, *in vitro* systems have long suggested that BCR engagement can directly induce cell death, reinforcing the idea that BCR inputs are not uniformly positive even when CD40 signaling is present. In CD40-preactivated murine splenic B cells, BCR stimulation induces apoptosis despite ongoing CD40 signaling, through the pro-apoptotic mediator Bim (32). Additionally, BCR cross-linking in human B cells has been shown to engage a caspase cascade, including caspase-8 activation upstream of mitochondrial permeabilization and subsequent caspase-9/-3 activation in a positive feedback loop (33), consistent with broader reviews that caspase pathway usage in B cells is context dependent (34). Moreover, BCR engagement also imposes a metabolic liability by driving sustained ROS production, coupled to mitochondrial dysfunction and apoptotic death (35–37). Together, these studies established BCR activation-induced cell death (AICD) as a phenomenon and point to the same cell-intrinsic apoptosis pathway as the relevant mechanism. However, the precise wiring downstream of the BCR remains obscure, motivating further intracellular models to identify the primary BCR-proximal adaptor that initiates the cascade (**Figure 1**).

Crucially, AICD is a dominant constraint on B-cell selection, since early GC clonal dynamics show that cell death is a major determinant of clone size, rather than affinity-dependent proliferation alone. Taylor et al. reported that the population size of each antigen-reactive clone showed no relationship to the fraction of cells that had undergone extensive division, in pooled splenic and lymph node B cells at 7 days post-immunization, implying that clone-to-clone differences in proliferative capacity do not determine the eventual clonal size (38). By contrast, this relationship is restored in Bim-deficient B-cell clones that are resistant to AICD, which do show significant correlation between the number of cells and cell division (38). Overall, this decoupling in normal physiology is conceptually important for understanding B-cell selection based on intracellular molecular network integration: since proliferation cannot account for clonal output, cell death must be treated as the primary variable when modeling how BCR and CD40 signals shape B-cell selection and tolerance.

Since cell death is recognized as a primary determinant of B cell clonal expansion, the next question is what controls how BCR engagement is converted into clonal survival or deletion. An emerging framework from recent advances is that this choice depends not only on BCR signal strength, but also on timing: strong BCR stimulation can initiate a time-evolving pro-apoptotic program that becomes increasingly difficult to reverse, while appropriately timed secondary signals (e.g., CD40L or CpG) can still restore viability (20, 21). In this framing, tolerance is not enforced by a static affinity cutoff, but by the intracellular molecular network dynamics, through a kinetic competition between the progression of the apoptotic cascade involving Caspase 8, caspase 3 and the mitochondrial feedforward loop, and CD40-driven induction of anti-apoptotic programs via NF-κB activation and Bcl2 family members and c-Myc, as captured in a dynamical model of BCR-CD40 signal integration (20) (**Figure 1**). This provides a mechanism by which self-reactive GC B cells could be selectively and proactively deleted when T cell help is absent, delayed, or limited, even if proliferation is eventually triggered. Overall, these studies reinforce the perspective that we must shift our conceptual framework from static affinity thresholds to the analysis of intracellular and intercellular network dynamics. If B-cell fate depends on the timing and availability of Tfh help relative to antigen sensing, the next question is which features of the GC environment control that timing *in vivo.*

## 3 Intercellular models of signals: the temporal relationship of antigen and T cell encounters

Given that B cells make fate decisions based on the temporal integration of BCR and CD40 signals, it is not surprising that the organization of the GC contains features that modulate the temporal relationship between antigen binding and access to T-cell help. These mechanisms do not always eliminate self-reactive T-cell help. Rather, they create a disparity in the probabilities of self- and foreign-reactive B cells to receive timely, productive rescue, thereby generating the bias that can later be amplified across successive rounds of light-zone selection and dark-zone proliferation.

**Figure 2:**
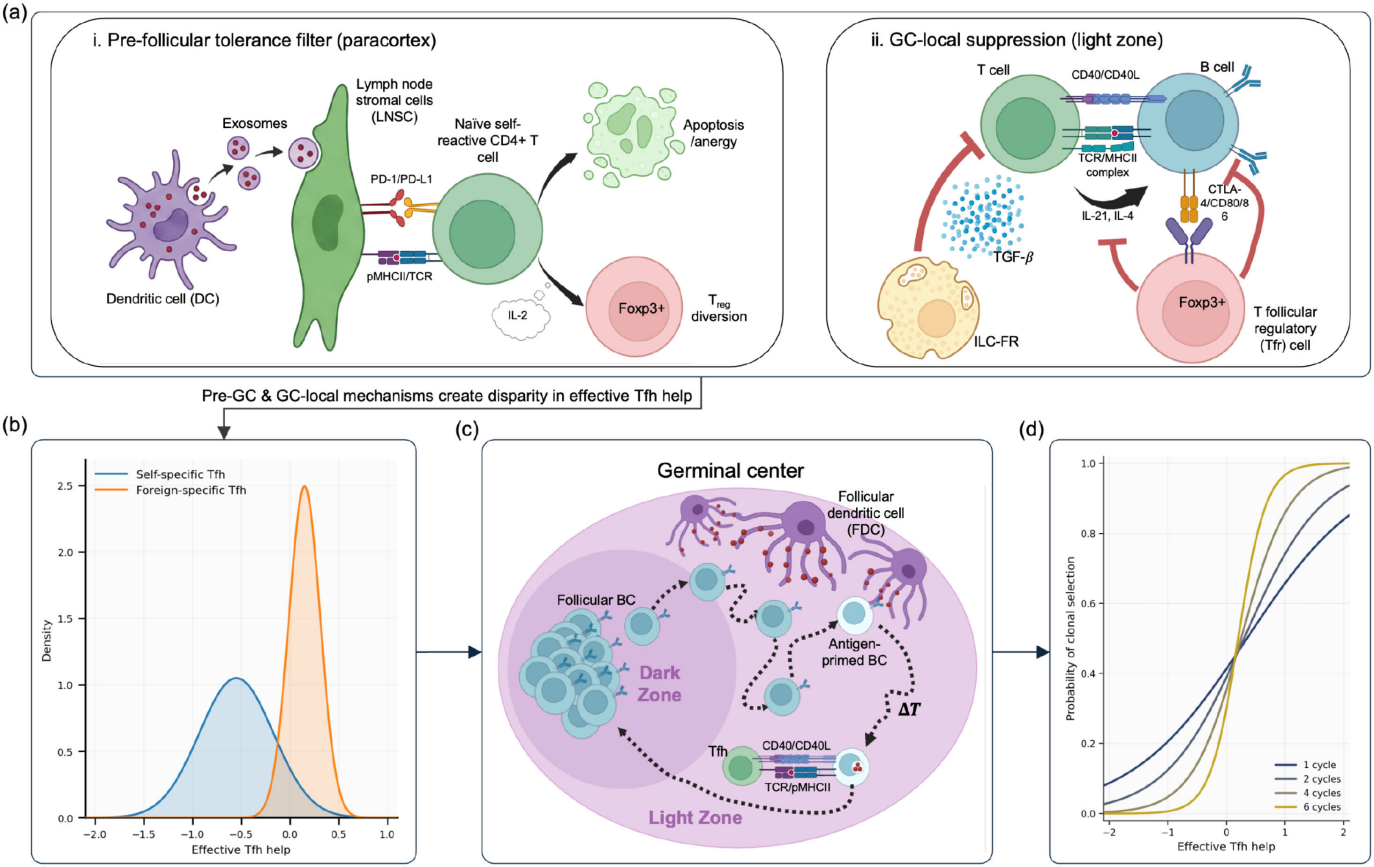
Intercellular mechanisms bias effective self-cognate Tfh help, and iterative GC cycling amplifies this bias into stringent clonal selection against self-reactive B cells. **(a)** Mechanisms for reducing self-cognate help. (i) Lymph-node stromal cells acquire and present self-peptide-MHCII (often via transfer from dendritic cells) in an inhibitory, low co-stimulation context, promoting deletion and/or dysfunction of self-reactive CD4 precursors and/or differentiation into regulatory fates. This reduces the pool of self-reactive helper cells available to enter follicles. (ii) Within follicles, regulatory populations (Tfr and ILC-FR) dampen Tfh help signals (e.g., via CTLA-4-dependent effects and TGF–*β*-mediated suppression), making productive help for self-linked interactions rarer and weaker. **(b)** Net effect on helper availability. The layered controls shift the distribution of effective Tfh Ahelp so that self-specific (blue) helper availability is diminished relative to foreign-specific (orange), i.e., self-cognate help is less frequent and/or less accessible. **(c)** Amplification during the GC reaction. GC selection-proliferation cycles repeatedly test B cells: cells must acquire antigen, present pMHCII, and win Tfh help in the light zone in a timely manner to earn proliferation in the dark zone. Iterating this cycle compounds small, persistent differences in help availability, progressively enriching clones that more reliably receive help. **(d)** Emergent clonal selection stringency across cycles. As small differences in effective Tfh help are propagated through repeated GC cycles, the relationship between Tfh help and probability of clonal selection becomes increasingly steep and switch-like. In this framework, reduced self-cognate Tfh availability makes self-reactive lineages disproportionately likely to fall below the effective help threshold over time, increasing their extinction probability.

The composition and organization of the GC constrain self-cognate Tfh help at multiple stages (**Figure 2a**). Prior to T cell differentiation and GC entry, lymph node stromal cells (LNSCs) impose a pre*-*follicular tolerance filter by shaping how naïve CD4 T cells experience self-antigen in the lymph node (39, 40). LNSCs acquire peptide-MHCII complexes from migratory dendritic cells (DCs) in a contact-dependent manner (potentially via DC-derived exosomes), then present these transferred complexes to naive CD4 T cells (**Figure 2ai**) (41). Critically, unlike professional antigen-presenting DCs, these stromal cells have low to absent expression of classical costimulatory molecules (CD80/86) but high inhibitory cues (PD-L1) (40, 41). Functionally, this attenuates self-reactive CD4 T cell responses, reducing their proliferation and sometimes promoting apoptosis (41). Because this interaction is specifically mediated by transferred pMHCII, it is intrinsically antigen-specific. Under homeostasis, this persistently diverts self-reactive T-cell precursors away from Tfh differentiation, without equivalently penalizing foreign-specific clones primed by activated APCs during inflammation (41). Complementarily, LNSCs can also drive antigen-specific differentiation into the regulatory T cell (Treg) fate in an IL-2-dependent manner, further repressing autoreactive Tfh differentiation and downstream GC responses against the same self-antigen (42).

Within follicles, multiple GC-local regulators can further trim effective self-reactive T-cell help by reducing the frequency and/or potency of productive cognate Tfh-B encounters (**Figure 2aii**). T follicular regulatory (Tfr) cells are a Treg-lineage population that localizes to follicles and germinal centers. Importantly, developmental and repertoire data indicate that Tfr cells derive primarily from thymic-educated precursors rather than the antigen-differentiated Tfh pool (43, 44). Hence, Tfh and Tfr cells within the same GC draw from largely distinct TCR repertoires, with evidence for enrichment of self/self-like specificities in the Tfr compartment (45, 46). Mechanistically, the best-defined pathway by which Tfr cells inhibit Tfh responses is CTLA-4-dependent suppression, which restrains antigen-specific Tfh expansion and therefore limits B-cell responses *in vivo* (47–49). Additional *ex vivo* evidence supports reduced Tfh cytokine output under Tfr influence (50, 51), notably IL-4, and to a lesser extent IL-21, which promote B cell proliferation and plasma differentiation, although context-dependent roles such as Tfr-derived IL-10 in supporting aspects of GC responses have also been reported (52, 53). In parallel, follicular regulatory innate lymphoid cells (ILC-FR) have been described as a tissue-resident ILC subset enriched in human lymphoid tissue, localized in the GC light zone (LZ) microenvironment where Tfh-B interactions occur (54). Functionally, in ex vivo Tfh/GC-B co-culture systems, ILC-FR suppress Tfh/GC-B co-culture outputs (reduced IgG and diminished IL-21 and soluble CD40L), with evidence pointing to robust TGF-*β* production as a major mediator (54).

Together, these pre-GC filters and GC-local repressive mechanisms provide convergent evidence that the GC organization reduces self-reactive Tfh availability, by lowering their precursor entry, limiting their expansion, and decreasing per-contact potency of their help signals, without requiring the complete elimination of self-reactive T cells (**Figure 2b**). This prolongs the time-gap between BCR and CD40 signals for self-reactive B-cells, thereby biasing them toward negative selection and thus enforcing peripheral tolerance. Once such intercellular regulation creates even a modest but persistent difference in the probability of timely Tfh help, the next question is how that bias is propagated through the cyclic dynamics of the GC reaction.

## 4 A multi-scale model of stochastic dynamics across iterative cycles of selection

A defining feature of the GC reaction is cyclic re-entry between spatially segregated light (LZ) and dark zones (DZ). B cells iteratively alternate between selection in the LZ, where they compete for Tfh help after antigen capture and peptide presentation, and expansion and mutation in the DZ where selected cells undergo several rounds of division and SHM before returning for further selection (**Figure 2c**). This LZ/DZ cycle is supported by intravital imaging and transcriptional/phenotypic separation of LZ- and DZ-like GC B cells, and is now a core organizing principle for affinity maturation (7, 55–58). In this framework, the GC does not decide self-versus-foreign in a single step, but repeatedly tests clones across multiple cycles. Differences in the likelihood of receiving Tfh rescue signals compound across cycles, producing large cumulative differences in time to dominance, peak clone size, and the probability of self-clone extinction (7, 18, 58) (**Figure 2d**). Crucially, this amplification only requires that GC-local regulation imposes a persistent, directional bias in the probability of productive Tfh help for self- versus foreign-derived antigens, after which cyclic re-entry applies a nonlinear gain through a series of amplifiers.

The first amplifier is engaged at the level of Tfh-B interaction dynamics. Intravital imaging showed that ICOSL on GC B cells promotes an entangled mode of Tfh-B interaction (59), where contacts are brief but involve extensive surface engagement, trigger productive Tfh Ca^2+^ spikes, and are associated with B-cell acquisition of CD40 help signals. ICOSL surface expression on GC B cells is upregulated by CD40 signals, creating a positive feedback loop that increases both the likelihood and quality of future Tfh-B contacts (59). Over time, repeated entanglement promotes the co-localization of more competitive GC B cells with Tfh cells near the outer LZ periphery close to the lymph node T-zone, which effectively gives the already advantaged clones improved access to T-cell help (59).

The second amplifier translates selection in the LZ into proliferative expansion in the DZ. This is mediated by a transcriptional program where Tfh help induces transient Myc expression in positively selected LZ B cells, and the magnitude of Myc relates to their subsequent proliferative capacity (60). Myc induction scales with antigen capture and help, and its amount determines both division capacity and residence time in the DZ, i.e., how much time a selected cell expands for before returning for another selection round (60). Together, Myc expression integrates the amount and quality of Tfh help accrued in the LZ and converts it into a programed number of B cell divisions in the DZ, allowing a greater number of daughter B cells to re-enter competition following one successful rescue event.

Recent work further sharpens the temporal logic of this integration. Kagan Ben Tikva et al. (61) showed that T cell-derived signals increase the frequency of Myc transcriptional bursts in GC B cells, rather than simply increasing transcript abundance per cell at a single time point. This implies that net Myc expression is a time integral of help-driven transcriptional bursting (61). In this view, a self-reactive clone does not just need one lucky cognate encounter; it needs repeated productive help at sufficient frequency to repeatedly re-initiate Myc bursts. Therefore, any decrease in self-reactive Tfh availability (fewer and/or less productive encounters) directly lowers the burst frequency of Myc expression and reduces the likelihood of engaging the B cell division program across successive cycles.

This burst-frequency framework also clarifies why the ICOS-ICOSL entanglement loop can be a potent amplifier. By promoting more frequent and reliably productive T-B interactions, and through CD40-dependent upregulation of ICOSL and preferential co-localization in Tfh-rich regions, ICOSL-mediated entanglement can selectively increase the frequency of help pulses for early B cell winners. This converts the initial disparities in access to cognate T cell help into larger disparities in integrated Myc exposure, DZ division output, and clonal expansion over time.

A potential concern with an amplifying selection program is the runaway, winner-take-all expansion of an unsuitable clone driven by noise. For selection to be robust, mechanisms need to be in place to stabilize against chance events that lead to any particular clone monopolizing help forever. The active turnover of peptide-MHCII (pMHCII) complexes displayed on GC B cells, particularly across the DZ phase, is one such mechanism. It ensures that pMHCII display on GC B cells is not the long-lived memory of a single successful antigen encounter or a single LZ cycle, and only reflects recent antigen acquisition (62). Thus, periodic targeting of pMHCII for ubiquitin-mediated degradation to favor presentation of newly acquired antigen promotes fidelity and efficiency of selection (62). This reset suppresses runaway amplifications from a single lucky encounter, while preserving the compounding of persistent differences between clones. Each cycle becomes an approximately independent test of a clone’s ability to (i) re-acquire antigen, (ii) generate competitive pMHCII, and (iii) secure Tfh help. In this way, only persistent disparities in the quality and accessibility of Tfh help are robustly amplified.

## 5 Conclusion

The original two-hit model for peripheral tolerance relies on the precision of removing autoreactive T-cell clones during thymic education and describes B-cell clonal expansion as a function of the affinity of the BCR to antigen. However, this framework is clearly incomplete. Neither is T-cell central tolerance sufficient, nor is antigen affinity the key determinant for clonal expansion. Moreover, BCR signaling itself exhibits seemingly paradoxical effects: while it is required for antigen capture and productive selection, strong BCR engagement can also promote apoptotic stress and death.

The resolution of the BCR signaling paradox, we argue, is that BCR engagement is neither intrinsically beneficial nor intrinsically deleterious. Rather, its outcome depends on the dynamic integration of signaling magnitude, duration, co-receptor context and most importantly, the timing of compensatory rescue relative to the progression of the BCR-induced pro-apoptotic program. Signal magnitude and duration shape how strongly and how rapidly pro-death or pro-survival programs accumulate, but whether a GC B cell survives or is deleted depends primarily on whether sufficiently productive Tfh-derived rescue, especially via CD40, arrives before the death program progresses into irreversibility.

In this view, the distinction between autoreactive and foreign-reactive clones rests on temporality across all biological scales. At the micro-scale within each B cell, the time delay between antigenic and Tfh-derived signals specifies negative versus positive selection. At the intermediate GC scale, tissue organization and regulatory cell composition shape the mean passage times for antigen uptake and access to Tfh help during the stochastic search by B cells, thereby biasing the probability of timely rescue. At the macro-scale of the overall response, iterative GC selection cycles compound these differences in rescue probability and amplify modest biases between clones into marked differences in clonal survival, expansion and extinction.

Understanding B-cell peripheral tolerance therefore becomes how temporally structured selection processes are generated, interpreted and amplified across scales. A multi-scale systems framework in which intercellular interaction dynamics are interpreted by an intracellular molecular network therefore provides a basis for understanding not only affinity maturation and clonal expansion, but also the active elimination of autoreactive B cells through negative selection.
